# Direct Thrombin Inhibitors Prevent Left Atrial Remodeling Associated With Heart Failure in Rats

**DOI:** 10.1016/j.jacbts.2016.05.002

**Published:** 2016-07-13

**Authors:** Céline Jumeau, Alain Rupin, Pauline Chieng-Yane, Nathalie Mougenot, Noël Zahr, Monique David-Dufilho, Stéphane N. Hatem

**Affiliations:** a Sorbonne University, Institut National de la Santé et de la Recherche Médicale (INSERM), Unité Mixte de Recherche 1166, Institute of Cardiometabolism and Nutrition (ICAN), Paris, France; b Servier Research Institute, Suresnes, France; c FRS Consulting, Paris, France; d Inserm-Sorbonnes-Universités, Unité Mixte de Service 28 Université Pierre et Marie Curie, Paris, France; e INSERM Assistance Publique-Hôpitaux de Paris, Groupe Hospitalier Pitié-Salpêtrière Department of Pharmacology and Centre d'Investigation Clinique 1421, Paris, France; f Cardiology Department, ICAN, Paris, France

**Keywords:** anticoagulant, atrial arrhythmia, direct thrombin inhibitor, heart failure, remodeling, AF, atrial fibrillation, ANP, atrial natriuretic peptide, BNP, brain natriuretic peptide, CTGF, connective tissue growth factor, DTI, direct thrombin inhibitor, MHC, myosin heavy chain, MI, myocardial infarction, NFATc3, nuclear factor of activated T cells 3, PAI, plasminogen activator inhibitor, PAR, protease-activated receptor

## Abstract

The present study tested the hypothesis that thrombin participates in formation of left atrial remodeling and that direct oral anticoagulants, such as direct thrombin inhibitors (DTIs), can prevent its progression. In a rat model of heart failure associated with left atrial dilation, we found that chronic treatment with DTIs reduces the atrial remodeling and the duration of atrial fibrillation (AF) episodes induced by burst pacing by inhibiting myocardial hypertrophy and fibrosis. In addition to the prevention of thromboembolism complicating AF, DTIs may be of interest to slow down the progression of the arrhythmogenic substrate.

## Highlights

• Oral direct anticoagulants such as DTIs, but not vitamin K antagonist, diminish the progression of atrial dilation associated with heart failure in a rat model.
• Prevention of atrial dilation by DTI is associated with decreased duration of atrial arrhythmia induced by burst pacing.
• DTI inhibits interstitial fibrosis, extracellular matrix remodeling, and hypertrophy of atrial myocardium.
• Plasma DTI concentrations inhibiting atrial remodeling are lower than those required for therapeutic anticoagulant activity.
• DTIs may be of interest to prevent the progression of atrial fibrillation substrate in addition to their anticoagulant activity.

## Visual Abstract

**Figure:**
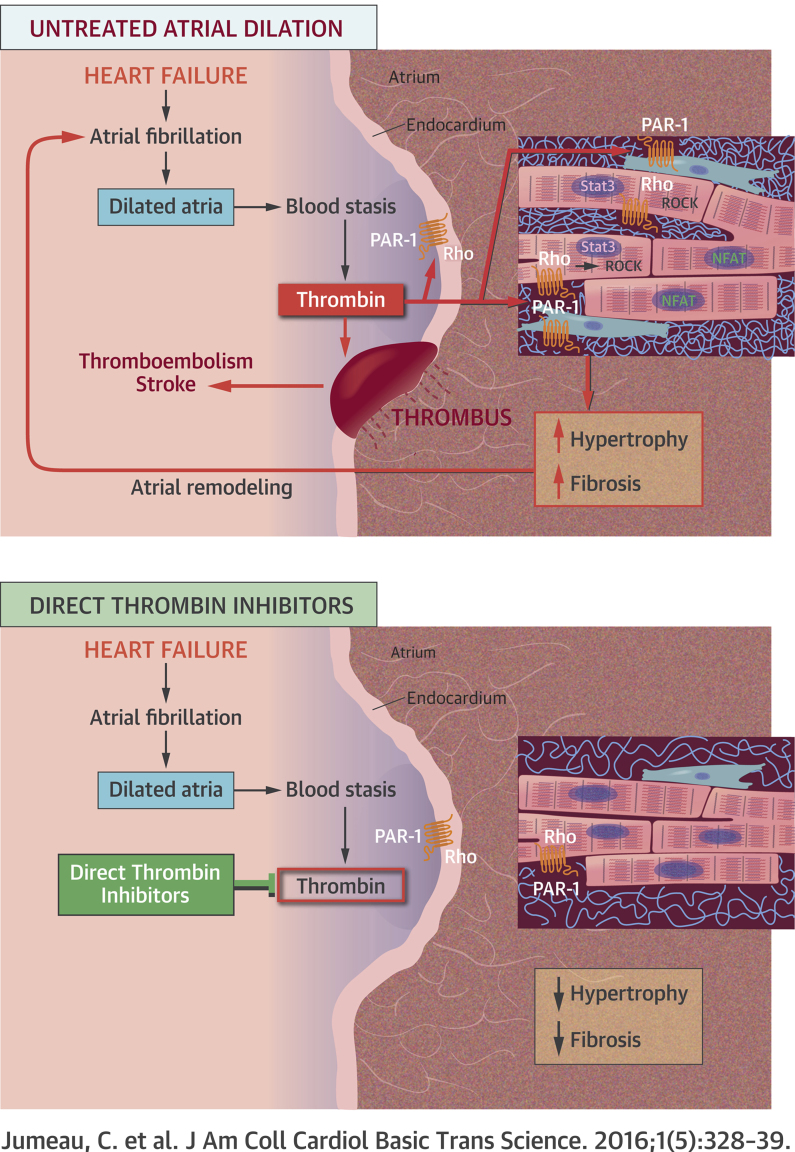

Atrial fibrillation (AF) is the most common sustained cardiac arrhythmia encountered in clinical practice (1). It is associated with a 5-fold risk of stroke and systemic thromboembolisms. During AF, thrombus formation is promoted by blood stasis in poorly contractile atria together with a hypercoagulable state, as indicated by high circulating levels of fibrinolytic degradation products, plasminogen activator inhibitor (PAI)-1, and thrombin-antithrombin complex (2). For all of these reasons, anticoagulation is a central therapeutic target for most AF patients. Anticoagulation can be achieved via vitamin K antagonists or, more recently, with direct thrombin inhibitors (DTIs) or direct factor Xa inhibitors, referred to as non–vitamin K antagonist oral anticoagulants (3).

Thrombin is the central protease of the coagulation cascade. It converts soluble plasma fibrinogen into insoluble clot-forming fibrin polymers, and activates several positive feedback steps to amplify its own generation (4). In addition, thrombin has pleiotropic cellular effects through the cleavage of protease-activated receptor (PAR)-1, including hemostasis, inflammation, cellular growth, and proliferation 4, 5, 6. For instance, PAR-1 promotes hypertrophy of neonatal rat cardiomyocytes and deoxyribonucleic acid synthesis in fibroblasts 5, 7. In mice, PAR-1 overexpression induces eccentric hypertrophy and dilated cardiomyopathy, whereas PAR-1 deficiency is associated with reduced left ventricle dilation after myocardial infarction (MI) (8).

Several hormones, peptides, or pathways are recognized to be involved in atrial remodeling, including the renin angiotensin system (9), but little is known about the role of thrombin. In vitro, this protein induces alterations of the electric and mechanical properties of rabbit left atrial strips, which are prevented by the DTI dabigatran and a PAR-1 antagonist (10). The present in vivo study was undertaken to test the hypothesis that thrombin participates in left atrial remodeling and AF substrate formation, known to be promoted by heart failure 11, 12, 13, and that DTI can slow their progression. It was conducted using a rat model of heart failure secondary to an extensive MI, which is associated with left atrial remodeling and AF susceptibility 14, 15. We found that DTIs and PAR-1 antagonists prevent atrial remodeling and reduce AF susceptibility.

## Methods

### Model of atrial remodeling following infarction-induced heart failure

This study had the approval of the local animal research ethics committee and the French Ministry of Education and Research (authorization N°00429.03). Male OFA Sprague-Dawley rats weighting 200 to 220 g were obtained from Charles River Laboratories (L'arbresle, France) and housed for 10 days before the surgery. Animals were anesthetized with intraperitoneal injection of 30 mg/kg sodium pentobarbital and received a subcutaneous injection of 1.5 mg/kg meloxicam for pain. MI was achieved by thoracotomy and transient occlusion of the left anterior descending coronary artery. After 30 min of ischemia, a definitive reperfusion phase was initiated. Sham rats underwent thoracotomy only. This model of heart failure was associated with a hypercoagulable state, as indicated by plasma thrombogenic potential assayed using calibrated automated thrombography (Appendix). The endogenous thrombin potential was similar at 5 and 56 days post-surgery for sham rats and was increased in rats with MI at the 3 times studied (Supplemental Figure 1).

### Treatments

Twice daily gavage with 12.5 mg/kg dabigatran etexilate or its vehicle (40% polyethylene glycol/60% H_2_O) and once-daily gavage with the PAR-1 antagonist F16618 (5 to 40 mg/kg) or its vehicle (1% methylcellulose) started 1 h post-MI, when rats regained consciousness. Warfarin was given orally in drinking water at 5 to 6.25 mg/l and 7.5 to 10 mg/l over 1 month, resulting in average doses of 0.43 and 0.64 mg/kg/day. Due to the high solubility of DTI S35972 in saline (vehicle), doses of 1.5 to 15 mg/kg/day were administered using ALZET osmotic minipumps (DURECT Corporation, Cupertino, California), which were subcutaneously implanted under anesthesia following the surgery. Rats were weighed before surgery, every week to adapt dabigatran or warfarin dosage, at 4 weeks post-MI to adapt S35972 dosage when changing the minipumps during anesthesia for echography, and at the end of treatment.

Detailed descriptions of all other experimental procedures are provided in the Appendix.

## Results

### DTIs inhibit atrial dilation and the development of arrhythmogenic substrate

**Figure 1:**
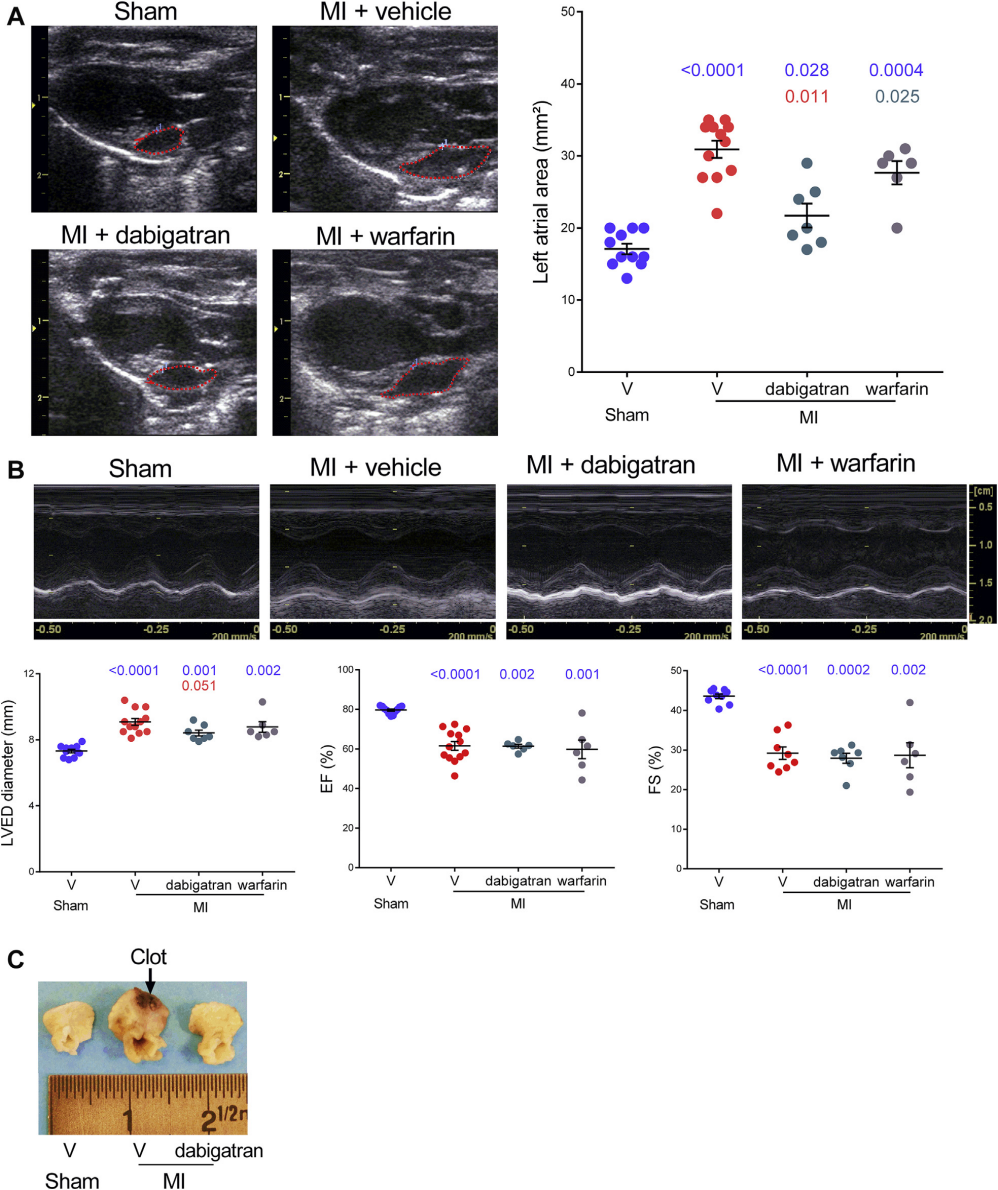
Dabigatran Etexilate Reduces Left Atrial Area in MI-Induced Heart Failure Sham rats and rats with myocardial infarction (MI) were treated over 4 weeks with 25 mg/kg/day dabigatran, 4.3 to 6.4 mg/kg/day warfarin, or vehicle (V). **(A)** Representative echocardiographic images in bidimensional mode and individual values of left atrial area. **(B)** Images in time-motion mode of left ventricular end-diastolic (LVED) diameters. **(C)** Representative pictures of left atria from sham rats and rats with MI at treatment end. n = 5 to 10 for each condition. Exact p values in **blue** are calculated versus sham, those in **red** versus vehicle-treated rats with MI, and the 1 in **gray** versus dabigatran-treated rats with MI.

To study the effect of DTIs on atrial remodeling, we first examined the effects of the oral DTI dabigatran etexilate, which has potent antithrombotic activity in rats in the range of 5 to 30 mg/kg (16). After 4 weeks of oral gavage, the surface of the left atria measured by bidimensional mode echocardiography increased 1.8-fold in vehicle-treated rats with MI compared with sham animals and only 1.3-fold in dabigatran-treated rats with MI (Figure 1A). Remarkably, treatment with the vitamin K antagonist, warfarin, did not significantly reduce left atrial dilation. Furthermore, dabigatran etexilate slightly reduced the left ventricular dilation without improving the systolic function evaluated by fractional shortening and ejection fraction (Figure 1B). The inhibitory effect of dabigatran on left atria size was confirmed by macroscopic examination at sacrifice of the animals (Figure 1C).

**Figure 2:**
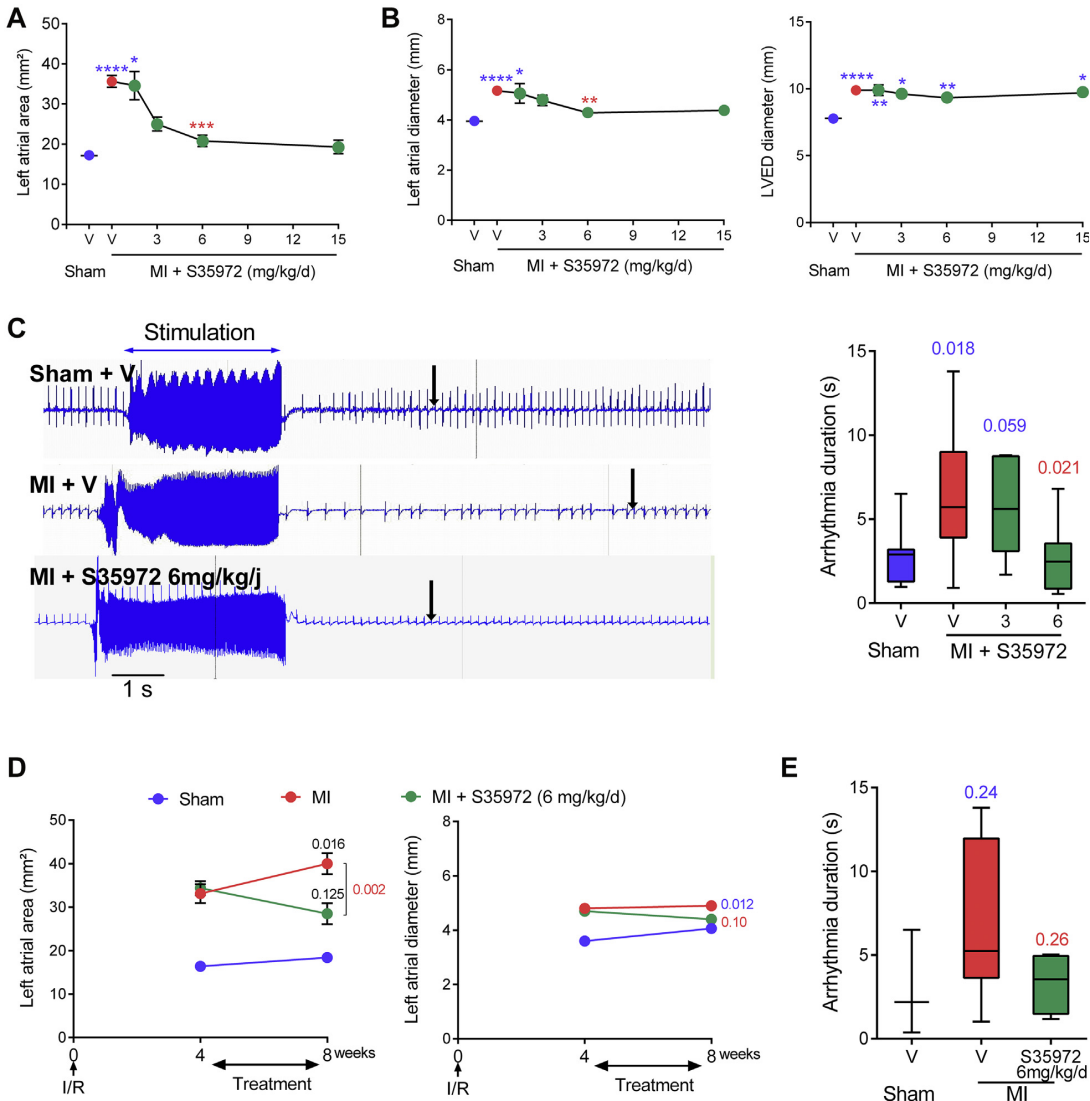
DTI S35972 Prevents Atrial Dilation and Reduces Arrhythmia Duration S35972 or vehicle were administered for 8 weeks. **(A)** Dose-dependent effect on left atrial area measured by bidimensional**-**mode echocardiography. **(B)** Measurements of left atrial and LVED diameters in time-motion mode. **(C)** Representative electrocardiographs and dose-dependent effect on pacing-induced atrial arrhythmia. The **arrow** indicates the return to sinus rhythm. **(D)** Measurements of left atrial area and diameter and **(E)** arrhythmia duration after 4 weeks of treatment, which started 4 weeks post-MI. **(A to C)** n = 5 to 15 for each condition. **(D to E)** n = 3 to 5, 8, and 4 to 6 for sham rats, vehicle-treated rats with MI, and S35972-treated rats with MI, respectively. *p < 0.05, **p < 0.01, ***p < 0.001, and ****p < 0.0001; exact p values calculated versus sham are in **blue**, and those versus vehicle-treated rats with MI are in **red**. Exact p values in **black** compared data before and after treatment. Abbreviations as in Figure 1.

Similar effects on atrial dilation were observed with another DTI, the nonprodrug S35972, which has anticoagulant and antithrombotic activities comparable to dabigatran etexilate between 1 to 20 mg/kg in rats 16, 17. In addition, because this drug is highly soluble, it could be subcutaneously delivered via osmotic minipumps, which is a less stressful and more reliable route of delivery than oral gavage. Thus, S35972 was used in the rest of the study. At 4 weeks post-MI, DTI S35972 dose-dependently reduced the left atrial area, which increased only 1.2-fold compared with sham animals at the optimal dose of 6 mg/kg/day, without any effect on left ventricle (Supplemental Figure 2A). As PAR-1 is the main thrombin receptor in the heart (5), we also tested the effect of the PAR-1 antagonist F16618 on atrial dilation. After 8 weeks of treatment, S35972 and F16618 dose-dependently diminished both left atrial size and left ventricular diameter without an effect on the ventricular function (Figures 2A and 2B, Supplemental Figures 2A and 2B). F16618 reduced the ventricular diameter at the optimal dose of 10 mg/kg/day (p = 0.004 vs. vehicle-treated rats with MI), whereas 6 mg/kg/day of S35972 had a little effect (p = 0.058). Furthermore, the duration of AF episode triggered by burst pacing was prolonged in vehicle-treated rats with MI but not in DTI-treated rats (Figure 2C).

Remarkably, the DTI S35972 also reduced the atrial dilation when the treatment started 1 month after the constitution of the cardiomyopathy. A total of 4 weeks of treatment inhibited the progression of the left atrial dilation. The atrial area increased 1.6-fold in DTI-treated animals compared with shams instead of 2.1-fold in vehicle-treated rats with MI (Figure 2D), with a trend of reduced average duration of AF from 6.75 s to 3.3 s (Figure 2E).

These results indicate that the DTIs inhibit and prevent the progression of the atrial remodeling associated with heart failure in rats.

### DTIs reduce atrial dilation independently of their anticoagulant properties

**Figure 3:**
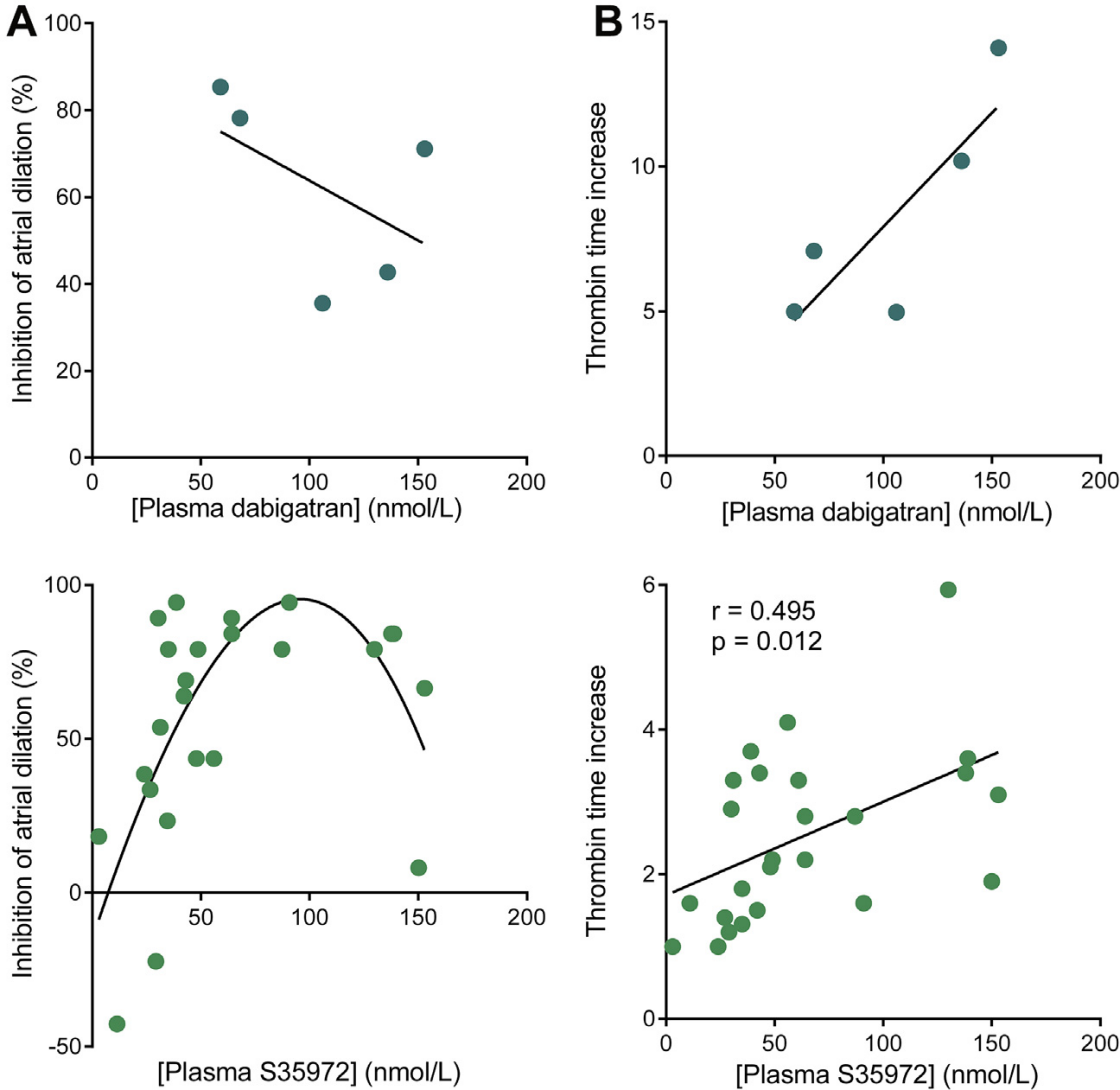
The Antiremodeling Properties of DTIs Are Independent of Their Anticoagulant Activity Treatment was with either 25 mg/kg/day dabigatran etexilate for 4 weeks or S35972 for 8 weeks at doses ranging 1.5 to 15 mg/kg/day. Direct thrombin inhibitor (DTI) plasma concentrations were assessed by liquid chromatography-tandem mass spectrometry. Inhibition of atrial dilation **(A)** and thrombin time increase **(B)** were calculated as described in the Appendix.

We next investigated the relationship between antiremodeling and anticoagulant properties of DTIs. First, we determined their availability in rat plasma by means of mass spectrometry. In rats with MI treated with 25 mg/kg/day dabigatran etexilate or 6 mg/kg/day S35972, the plasma concentrations ranged from 28 to 72 ng/ml at peak for dabigatran and 22 to 85 ng/ml for S35972, which corresponds to the low therapeutic levels of DTI detected in humans (18). The inhibition of atrial dilation was maximal at DTI concentrations ranging from 30 to 100 nmol/l as previously reported for the inhibition of thrombin-mediated PAR-1 cleavage by dabigatran (19), but it decreased with high plasma concentrations (Figure 3A). At the opposite end, the anticoagulant activity assessed by thrombin times increased with DTI plasma concentrations (Figure 3B). These results suggest that the antiremodeling effects of DTI are independent of their anticoagulant activity.

### DTI inhibits remodeling of atrial extracellular matrix

**Figure 4:**
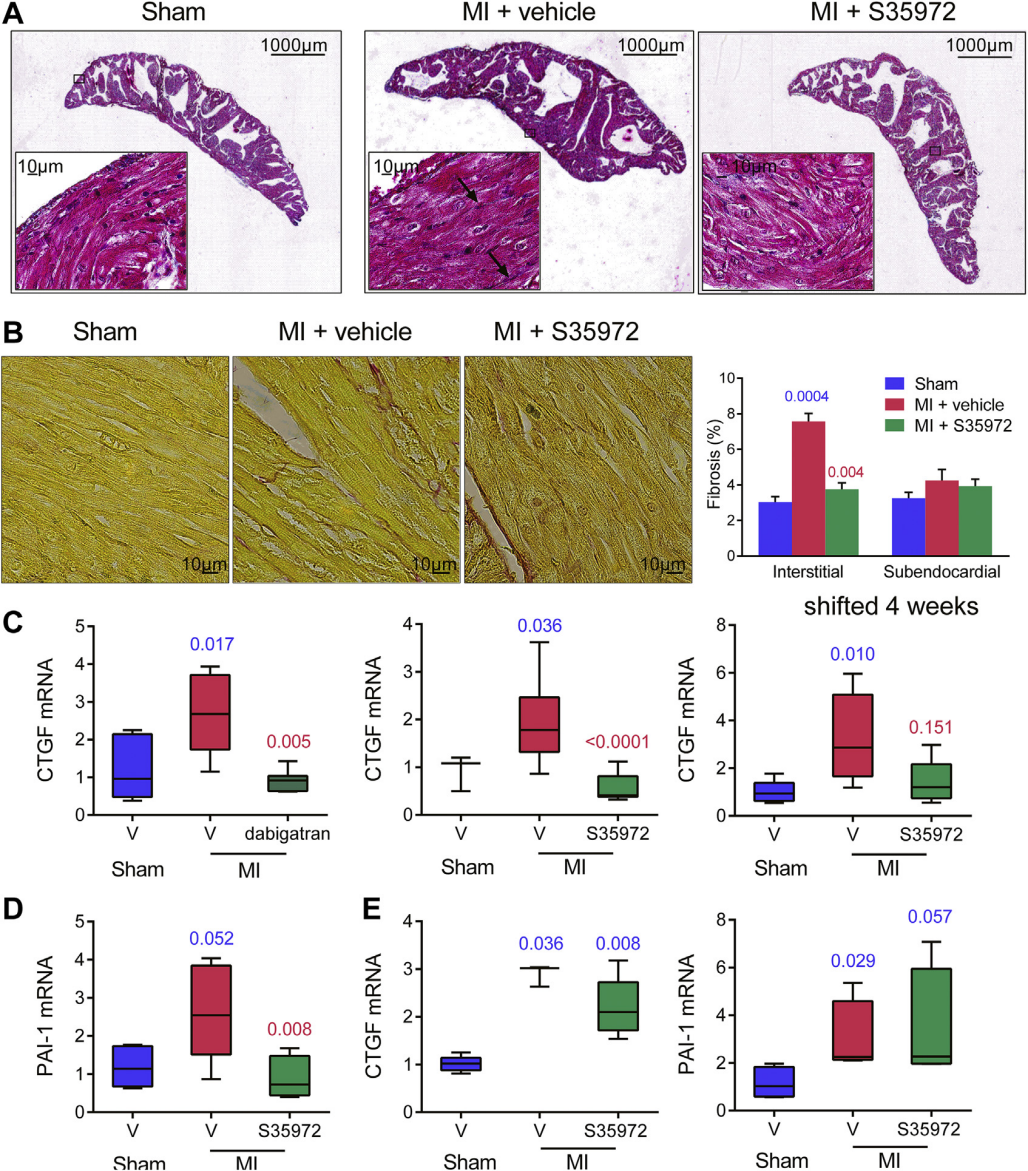
DTIs Reduce Myocardial and Matrix Remodeling in Left Atria Rats with MI were treated with vehicle or 25 mg/kg/day dabigatran etexilate for 4 weeks or 6 mg/kg/day S35972 for either 8 or 4 weeks, with initiation of treatment 1 month after MI (shifted 4 weeks). **(A)** Masson trichrome or **(B)** picrosirius red staining of atrial sections. Representative images were from the same rat in each group (n = 5 to 7). Magnification was ×100 and ×600. Atrial **(C and D)** and ventricular **(E)** messenger ribonucleic acid (mRNA) of connective tissue growth factor (CTGF) and plasminogen activator inhibitor 1 (PAI-1) were measured by real-time polymerase chain reaction. n = 3 to 11; exact p values in **blue** are calculated versus sham rats and those in **red** versus vehicle-treated rats with MI. Abbreviations as in Figures 1 and 2.

We next examined the histological determinants of the effect of DTI on atrial remodeling. Masson trichrome staining of atrial cross sections from vehicle-treated rats with MI revealed an overall hypertrophy of the myocardium, with enlarged myocytes and interstitial fibrosis as indicated by arrows in Figure 4A. In atria from rats with MI treated with DTI for 8 weeks, myocyte hypertrophy was markedly reduced and fibrosis was barely detectable. Staining of subendocardial and interstitial collagens with picrosirius red confirmed an increase in interstitial fibrosis in vehicle-treated rats with MI and a significant decrease in DTI-treated rats with MI (Figure 4B). We then analyzed the effect of DTIs on messenger ribonucleic acid (mRNA) expression using rat gene-specific primers (Supplemental Table 1). First, we studied mRNA expression of connective tissue growth factor (CTGF), which is involved in the remodeling of extracellular matrix (20) and is up-regulated in AF patients (21). Post-MI treatments for 1 and 2 months prevented the up-regulation of CTGF in left atria, and treatment initiated 1 month after the MI tended to decrease mRNAs (Figure 4C). We also analyzed the expression of PAI-1, which has both thrombogenic and fibrogenic activities 2, 20. Post-MI treatment for 2 months abolished the induction of PAI-1 in the left atria (Figure 4D), but it had no effect on both PAI-1 and CTGF mRNAs in the left ventricle (Figure 4E). These observations suggest that DTIs are able to prevent the extracellular matrix remodeling in the left atria but not in the left ventricle.

### DTI prevents atrial myocardial hypertrophy, as does PAR-1 antagonist

**Figure 5:**
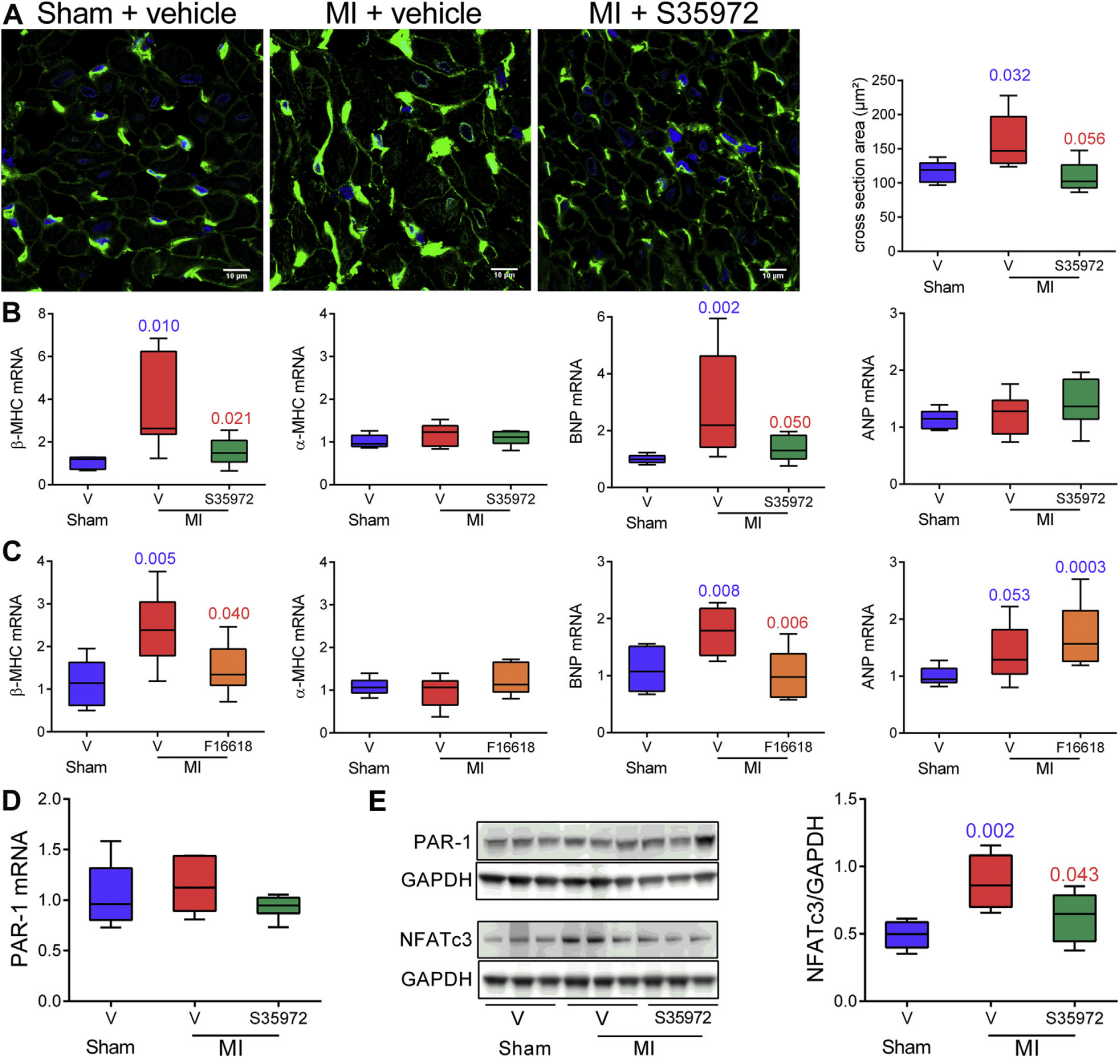
Thrombin and PAR-1 Contribute to Atrial Dilation By Stimulating Atrial Myocardium Hypertrophy Treatment with 6 mg/kg/day S35972, 10 mg/kg/day F16618, or vehicle lasted 8 weeks. **(A)** Immunofluorescence images and quantification of left atrial cross sections stained with 4',6-diamidino-2-phenylindole **(blue)** and wheat germ agglutinin **(green)**. **(B to D)** Real-time polymerase chain reaction analysis of relative mRNA expression for β- and α-myosin heavy chain (MHC), brain natriuretic peptide (BNP) and atrial natriuretic peptide (ANP), and protease-activated receptor-1 (PAR-1). **(E)** Immunoblots of PAR-1 and nuclear factor of activated T cells 3 (NFATc3) and densitometric analysis. n = 5 to 14 for each group; exact p values in **blue** are calculated versus sham rats and those in **red** versus vehicle-treated rats with MI. Abbreviations as in Figures 1 and 3.

We further characterized the antihypertrophic effect of DTI by measuring myocyte diameters in left atrial cross sections labeled with wheat germ agglutinin to stain plasma membrane. As illustrated in Figure 5A, atrial myocyte diameter increased 1.4-fold in the atria from vehicle-treated rats with MI compared with atria of S35972-treated and sham rats. Myocardial hypertrophy is associated with the reappearance of fetal phenotype including the expression of β-myosin heavy chain (MHC) and natriuretic peptides (22). At 8 weeks post-MI, β-MHC transcripts significantly increased, whereas the α isoform (α-MHC) did not change in dilated atria of vehicle-treated rats (Figure 5B). In addition, the mRNA level of brain natriuretic peptide (BNP) was increased, but not that of atrial natriuretic peptide (ANP). In sharp contrast, there was no change in β-MHC and BNP in atria from rats with MI treated with either the DTI S35972 or the PAR-1 antagonist F16618 in comparison with sham animals (Figures 5B and 5C). Of note, S35972 also down-regulated the expression of these markers in the ventricles of rats with MI (Supplemental Figure 3). We next analyzed whether PAR-1 expression was increased in rats with MI. Levels of both mRNA and protein were similar in the 3 groups of rats (Figures 5D and 5E), confirming activation of PAR-1 signaling in dilated atria. Among the transcription factors activated by PAR-1 (23), the nuclear factor of activated T cells 3 (NFATc3) is involved in maladaptive hypertrophy associated with heart failure (24) and thrombin-induced gene expression (25). DTI treatment prevented an MI-induced increase in the active form of NFATc3 (Figure 5E). These results suggest that the hypertrophic remodeling of atrial myocardium is partly due to an over activation of PAR-1 by thrombin.

### Thrombin and PAR-1 trigger hypertrophic signals via the Rho/Rho kinase pathway

**Figure 6:**
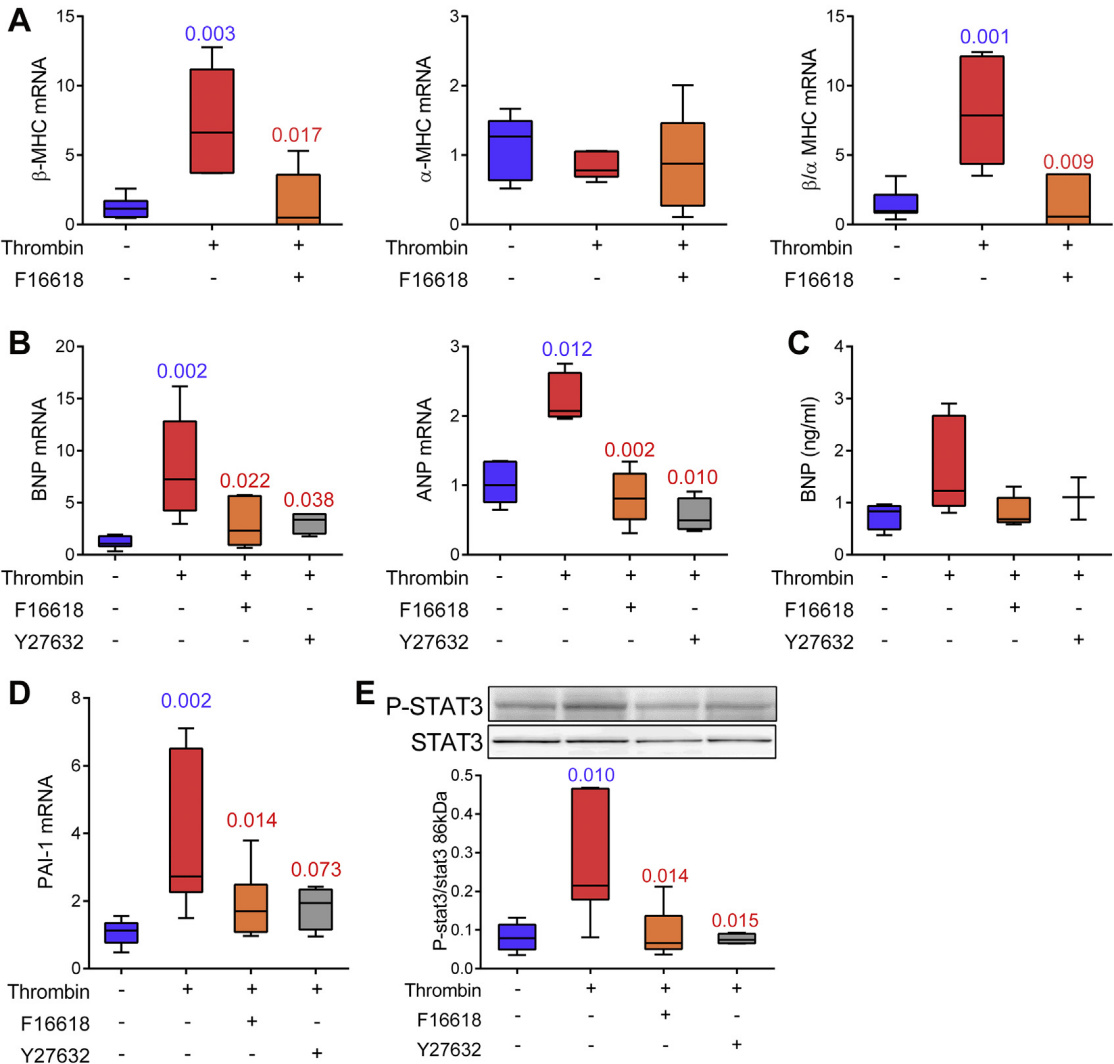
Rho Kinase and Transcription Factor Stat3 Are Involved in Thrombin-Mediated Hypertrophic Signals Left atrial explants were cultured for 7 days. Real-time polymerase chain reaction analysis of relative mRNA expression for β and α isoforms of MHC **(A)**, BNP and ANP **(B)** and PAI-1 **(D)**. **(C)** Quantification of soluble BNP by enzyme-linked immunosorbent assay. **(E)** Immunoblots of phosphorylated (P-) and total stat3. n = 3 to 7 for each condition; exact p values in **blue** are calculated versus untreated atria and those in **red** versus thrombin-stimulated atria. Abbreviations as in Figures 4 and 5.

To confirm the direct role of thrombin in atrial hypertrophy, we investigated its action in left atrial explants from control rats. This ex vivo model eliminated the possibility that mediators other than thrombin participate in the in vivo effects of DTI or PAR-1 antagonist. After 7 days of stimulation with 10 nmol/l thrombin, β-MHC but not α-MHC mRNAs were up-regulated, and this induction was prevented by F16618 (Figure 6A). Moreover, thrombin enhanced the expression of BNP and ANP transcripts and the release of soluble BNP through PAR-1 (Figures 6B and 6C). It also increased PAI-1 mRNAs (Figure 6D). Among the various PAR-1 effectors (23), the Rho-associated protein kinase (ROCK) is known to be involved in thrombin-induced endothelial permeability (26) and cardiomyocyte growth (27). Addition of the ROCK inhibitor Y27632 together with thrombin down-regulated BNP, ANP, and PAI-1 and reduced BNP secretion (Figures 6B to 6D). ROCK is a downstream effector of RhoA, which also activates the signal transducer and activator of transcription 3 (Stat3) (23). As expected, Stat3 is phosphorylated upon thrombin exposure, and both PAR-1 antagonist and ROCK inhibitor prevented this activation (Figure 6E). Altogether, these results indicate that thrombin/PAR-1 induce atrial hypertrophic phenotypes through the Rho cascade.

## Discussion

The major finding of the present study is that chronic thrombin inhibition with DTIs prevents the atrial remodeling and the AF susceptibility associated with heart failure in a rat model independently of their anticoagulant properties. This thrombin effect on atrial myocardium is mediated by PAR-1 signaling pathways, which are involved in the hypertrophy and fibrosis of atrial myocardium.

The inhibition of the atrial remodeling observed with DTI and PAR-1 antagonist cannot be attributed to the improvement of left ventricle function. Indeed, only a reduction of left ventricular end-diastolic diameter without recovery of systolic function was observed in treated animals. First, the large fibrotic scar of the infarcted left ventricle could have precluded any significant improvement of left ventricular function. Second, it has been shown that once established, atrial remodeling is no more sensitive to hemodynamic conditions in this model (9). However, PAR-1 deletion is associated with reduced left ventricle dilation and less impairment of ventricular function after MI in a PAR-1-knockout mouse line. This discrepancy could be due to the fact that PAR-1 deletion suppresses all the signaling pathways of the receptor, whereas DTI and antagonist block PAR-1 activation only by serine-proteases. Indeed, PAR-1 is activated by thrombin and other coagulation factors, but also by the matrix metalloproteinases at noncanonical sites (6).

### Thrombin inhibition prevents the development of atrial hypertrophy and fibrosis

The effects of DTI and PAR-1 antagonist on the atrial remodeling of rats with MI appears to be due to the inhibition of both myocardial hypertrophy and fibrosis. At the molecular level, both treatments normalize the BNP expression and β-MHC/α-MHC ratio, which are hallmarks of the reprogramming fetal gene program in diseased atria (22). DTI also decreased the levels of active NFATc3. In addition, the hypertrophic effect of thrombin on atrial myocardium was demonstrated ex vivo independently of the other neuro-mediators and hormones activated during heart failure. In left atrial explants, thrombin stimulated the expression of β-MHC, BNP, and PAI-1 through PAR-1 and the Rho/ROCK/Stat3 pathway. Our results are consistent with in vitro studies that showed stimulation by thrombin of hypertrophic cardiomyocyte growth and BNP production through PAR-1 and Rho cascade 7, 27. They also agree with a previous report of an increased nuclear accumulation of active NFATc3 and re-expression of hypertrophic genes in atrial myocardium from patients with chronic persistent AF (28). Furthermore, our observations that DTIs decrease interstitial fibrosis and atrial expression of PAI-1 and CTGF are consistent with a previous study showing a reduction of CTGF and pulmonary fibrosis by dabigatran (29). It is known that thrombin/PAR-1 stimulate the calcineurin/NFATc and RhoA pathways (23). Both are involved in cardiac hypertrophy and heart failure 24, 27, and RhoA regulates the endothelial permeability (26). During heart failure, thrombin activates the RhoA signaling to stimulate its extravasation through the endocardium and to induce remodeling of atrial myocardium (Visual Abstract).

### Dual antiremodeling and anticoagulant properties of DTIs

In the present study, the anticoagulant activity of DTIs was directly correlated to their plasma concentrations, whereas their antiremodeling effect was maximal between 35 to 100 nmol/l and decreased at higher concentrations. Furthermore, we showed that 10 nmol/l thrombin, a concentration reached during the initiation phase of coagulation cascade (30), was sufficient to induce hypertrophic genes in atrial myocardium in vitro. Interestingly, maximal inhibition of PAR-1 cleavage is obtained in vitro with 30 to 100 nmol/l dabigatran and 10 nmol/l thrombin, whereas higher DTI concentrations have activating effects (19). It is known that PAR-1 is activated at subnanomolar thrombin concentrations (31). Altogether, these data suggest distinct dose-dependency between the anticoagulant and antiremodeling effects of the DTIs. Indeed, they could inhibit the cellular actions of thrombin via PAR-1 before activation of coagulation cascade and formation of intra-atrial thrombus. In dilated and fibrillating human atria characterized by a high risk of thrombus, it is likely that such a low level of thrombin is reached. In this line, DTI plasma concentrations, which inhibit atrial dilation in rats (28 to 75 ng/ml at peak and 22 to 85 ng/ml at steady-state) correspond to the low therapeutic levels in humans. For example, DTI plasma levels, which have been measured in the RE-LY (Randomized Evaluation of Long-Term Anticoagulation Therapy) trial, ranged from 2 to 1,000 ng/ml at peak and 1 to 809 ng/ml at steady-state for the high dose of 150 mg dabigatran etexilate twice daily (18).

### Study limitations

The relevance of our experimental data to the human AF cannot be firmly established. They were obtained in a model of atrial remodeling, which develops with HF but not with spontaneous atrial arrhythmias. In some samples of human right atria from patients with paroxysmal AF, dilated atria, and normal ejection fraction (Supplemental Table 2), we analyzed mRNA expression of PAI-1 and hypertrophic genes using human gene-specific primers (Supplemental Table 3). PAI-1 and β-MHC/α-MHC ratio were increased, whereas the anticoagulant thrombomodulin was decreased (Supplemental Figure 4). This suggests a potential thrombin accumulation in dilated atria, because thrombomodulin accelerates its degradation (32). This is also consistent with a previous study on human atria that reported PAI-1 induction by the coagulation factor Xa in combination with rapid pacing of human atrial slice (33). Clinical studies on DTI have mainly evaluated the frequencies of stroke and major bleeding, and as specified for the large RE-LY trial, the case report forms were not prospectively designed to collect echocardiogram details (34). The current RE-CIRCUIT (Randomized Evaluation of dabigatran etexilate Compared to warfarIn in pulmonaRy vein ablation: assessment of different peri-proCedUral antIcoagulation sTrategies) (35) and COMMANDER HF (A Study to Assess the Effectiveness and Safety of Rivaroxaban in Reducing the Risk of Death, Myocardial Infarction or Stroke in Participants With Heart Failure and Coronary Artery Disease Following an Episode of Decompensated Heart Failure) trials (36) should help answer the question of whether DTIs can reverse cardiac remodeling in humans. RE-CIRCUIT evaluates the use of uninterrupted dabigatran etexilate during ablation procedures in patients with permanent or paroxysmal AF (35). COMMANDER HF assesses the efficacy of a low dose of direct factor Xa inhibitor in reducing all-cause mortality in HF patients with coronary artery disease (36). Finally, we provided no information on the exact mechanism by which DTIs reduce arrhythmia duration in our model. The antifibrillatory effect of DTIs might be explained by the effect of thrombin on cardiac ion currents, such as the activation of late component of sodium current in atrial myocytes 37, 38.

## Conclusions

There is a strong drive to understand mechanisms underlying the aggravation of arrhythmia and the transition from paroxysmal to permanent AF. The present study provides the first evidence for a role in vivo of thrombin/PAR-1 signaling in the development of atrial dilation and arrhythmogenic substrate. Our finding that thrombin contributes to the aggravation of atrial hypertrophy and fibrosis points out a novel potential therapeutic application for DTIs that is independent of their anticoagulant activity.

Perspectives **COMPETENCY IN MEDICAL KNOWLEDGE:** AF is associated with a high risk of systemic thromboembolism due to blood stasis in poorly contractile atria and a hypercoagulable state. Hence, most AF patients are under anticoagulant therapies. In an animal model, DTIs, including dabigatran, prevent or even reverse the atrial remodeling and AF susceptibility associated with heart failure by reducing atrial remodeling. This PAR-1–mediated effect is independent of anticoagulation properties of DTI.

**TRANSLATIONAL OUTLOOK:** The ongoing COMMANDER HF trial investigates whether targeted reduction of thrombin generation may improve clinical outcomes of patients with heart failure and coronary artery diseases. Its results should affirm or invalidate the clinical translation prospects of our observations in a rat model of heart failure-associated atrial remodeling.
