# Supplementary material for: Direct Thrombin Inhibitors Prevent Left Atrial Remodeling Associated With Heart Failure in Rats
Source: JACC Basic Transl Sci. 2016 Jul 13;1(5):328–39. doi: 10.1016/j.jacbts.2016.05.002 (PMC5012373; doi:10.1016/j.jacbts.2016.05.002)
Supplement: Supplemental Data [file mmc1.pdf]

ONLINE APPENDIX

Detailed Methods

Materials: The direct thrombin inhibitors (DTIs), Dabigatran etexilate (Ethyl N-[(2-{[(4-{N-[(hexyloxy)carbonyl]carbamimidoyl}phenyl)amino]methyl}-1-methyl-1H-benzimidazol-5-yl) carbonyl]-N-2-pyridinyl-β-alaninate) and S35972 (dihydrochloride salt of 4-oxo-3-(2-pyridin-2-ylethylamino)-4,6,7,8-tetrahydro-pyrrolo[1,2-a]pyrazine-6-carboxylic acid 2-ethylcarbamoyl methoxybenzylamide) were synthesized by Servier Research Institute (Suresnes, France) (1). The protease activated receptor 1 (PAR-1) antagonist, F16618 (2-[5-oxo-5-(4-pyridin-2-ylpiperazin-1-yl)-penta-1,3-dienyl]-benzonitrile hydro-chloride) was synthesized at the Pierre Fabre Research Institute (Castres, France) as previously described (2). Rat thrombin was purchased from Sigma-Aldrich (St. Louis, MO) and Rho kinase inhibitor, Y27632 from R&D systems (Lille, France). Dabigatran and $^{13}C_6$-dabigatran, used as internal standard (IS), were purchased from ALSACHIM® (Strasbourg, France). Liquid Chromatography-MS/MS grade water and methanol hypergrade for LC-MS Lichrosolv were purchased from MERCK® (Darmstadt, Germany). Formic acid hypergrade for LC-MS and dimethylsulfoxide (DMSO) of analytical grade were respectively purchased from VWR-PROLABO® (Fontenay-sous-bois, France) and from Sigma-Aldrich.

Surgical model of myocardial infarction (MI): Animals were anesthetized with 30 mg/kg sodium pentobarbital, and then intubated and ventilated at 60 respirations/min while body temperature was maintained at 37°C on a heating pad (Harvard Apparatus, Holliston, MA). Before the surgical procedure, they received a subcutaneous injection of 1.5 mg/kg metacam for pain. As previously described, rats underwent left thoracotomy and their left coronary artery was either left intact (sham) or occluded with a 4.0 silk suture at 1 mm from its origin to cause myocardial infarction (MI). Ischemia was maintained for 30 min and the clamp was removed to initiate a definitive reperfusion phase (3). Rats were weighed before surgery and at the end of each treatment.

Transthoracic echocardiography: Cardiomyopathy development in rats was monitored under 1.5-2% isoflurane anesthesia by transthoracic echocardiography as previously described, using a Vivid 7 dimension cardiovascular ultrasound system equipped with a probe of 9-14 MHz frequency (GE Healthcare, Vélizy, France) (4). Bi-dimensional (B) and time-motion (TM) mode views were recorded along the parasternal long axis from mitral and aortic valves to apex and in a short axis at the level of the mid papillary muscles. Left atrial area (LAA) was measured using B-mode. TM-mode was used for measurements of left atrial diameter and left ventricle parameters such as diameter (Vd) and volume (Vd) at end diastole, diameter (Ds) and volume (Vs) at end systole. All measurements were performed and averaged over five consecutive cardiac cycles by one single blinded operator. Reproducibility was assessed by triplicate measurements.
The percentage of left ventricular fractional shortening (FS) in the long-axis view was calculated as (Dd – Ds)/Dd x 100. The left ventricle ejection fraction percentage (EF) was calculated as (Vd-Vs)/Vd x 100. The inhibition of atrial dilation by DTI was calculated as (Mean LAA of vehicle-treated MI group – LAA of DTI-treated MI-rat)/mean increase x 100. The increase in LAA was calculated as (LAA of vehicle-treated MI rat – mean LAA of sham group).

Paroxysmal atrial fibrillation episodes in rats
Atrial fibrillation episodes were induced by atrial burst pacing at the end of DTI treatment. Under anesthesia, a 4-French stimulation catheter (Saint Jude medical) was inserted into esophagus to the level of the left atria. Left atria were then stimulated for 3 sec at 10 Hz frequency and an ECG was recorded. AF duration was calculated as the time between the end of stimulation and recovery of sinus rhythm.

Blood and tissue sampling in rats: At the end of treatment, rats were anesthetized with 160 mg/kg sodium pentobarbital and 5 mL of blood was collected by cardiac puncture using citrate-coated 19 G

needle and syringe and transferred into a citrate-coated vacutainer (Terumo, Guyancourt, France). Immediately after blood sampling, hearts were harvested and weighed. Left atria were rapidly dissected and weighed. Right ventricles were discarded and left ventricles with septum were weighed. Both left atria and ventricles were divided in two and frozen in liquid nitrogen either directly for PCR and western blot analysis or after fixation in embedding medium for histology and immunofluorescence analysis. These tissue samples were stored at -80°C until analysis.

Thrombin generation tests

After sampling, blood was rapidly centrifuged twice for 10 minutes at 2,500 g and 20°C, then plasma samples were stored at -80°C.

The plasma thrombogenic potential was assessed using calibrated automated thrombography (Thrombinoscope BV, Maastricht, Nederlands) as previously described (5). The endogenous thrombin potential, which reflects the total activity of thrombin during coagulation, is the area-under-the-curve of thrombograms.

Determination of thrombin time and plasma DTI levels

Blood samples were centrifuged once for 10 minutes at 2,500 g and 20°C.

The anticoagulant activity of DTIs was evaluated within three hours of blood sampling by measurements of the thrombin time, which is exquisitely sensitive to dabigatran (6). Thrombin time measures the rate of clot formation in plasma after addition of exogenous thrombin. For dabigatran, blood sampling was performed 1h30 after the last gavage. Thrombin time was assessed using the STA thrombin kit (Diagnostica Stago, Asnières, France) according to the manufacturer's instructions. Increases in thrombin time were calculated in comparison to the mean value of sham rats.

For detection of DTIs levels, plasma were stored at -80°C. DTIs were quantified by high performance liquid chromatography-tandem mass spectrometry using an Acquity ultra performance liquid chromatograph system coupled to a triple quadrupole mass spectrometer from Waters® (France) as previously described (7). A single-step protein precipitation was used for plasma sample preparation. The analytes were detected in multiple reaction monitoring mode (MRM) using positive ionization mode. Quantification transitions were 472.2/→289.1 m/z and 478.3→295.1 m/z for dabigatran and internal standards. The lower limit of quantification was 2.5 ng/mL. The plasma concentrations were calculated with a molecular weight of 471.51 g for dabigatran and 536.5 g for S35972.

Atrium organ culture model

Cultures of cardiac atrial explants were performed as previously described (8). Left atria from male OFA Sprague-Dawley rats weighting 440-480g were isolated from the whole heart and dissected out from the aperture of the mitral valve. The septal parts were discarded and only the trabeculated myocardium walls were conserved and placed in transwell inserts (Corning Life Sciences, Hazebroucke, France), with endocardium facing the porous membrane (0.4 µm pore size). The culture wells containing medium 199 (Life Technologies, Carlsbad, CA) with 1 nmol/L isoproterenol, 0.1 % insulin transferrin selenium, 10 mmol/L glucose, Penicillin/Streptomycin and 10% fetal calf serum. The atrial explants were cultured for seven days with daily media changes, in either the absence or presence of 10 nmol/L thrombin alone, or in combination with 10 µmol/L F16618 or 10 µmol/L Y27632. At the end of culture, conditioned media were collected and atria were rapidly frozen in liquid nitrogen and stored at -80°C for subsequent analysis. Media were centrifuged for 15 minutes at 14,000 g to remove particles and supernatants were then stored at -80°C until quantification of soluble proteins.

Quantification of soluble BNP

Secretion of brain natriuretic peptides (BNP) from rat atrial explants in culture was assessed with an ELISA kit for BNP-45 from Innovative research (Sarasota, FL).

Reverse transcription and quantitative polymerase chain reaction

Total RNAs were extracted with TRIzol® according to the manufacturer's instructions (Life technologies). Contaminating DNA was removed by DNAse I treatment, amount of extracted RNA

was determined via the Nanodrop apparatus (Thermo Fisher Scientific, Illkirch, France) and its quality and purity was assayed by migration on 1% agarose gels. cDNA was synthesized from 500 ng total RNA by reverse transcriptase-polymerase chain reaction (RT-PCR). Real time qPCR was performed with 5 µL of cDNA diluted 1/10 as previously described (9). The specificity and efficiency of each primer set was monitored by analyzing dissociation and standard curves. For each gene, real-time RT-PCR was normalized to three internal control genes *RPL32*, *RPLP0* and *PolR2a* (10). The gene-specific primers used in the present study are listed in Online table 1 for rat and table 3 for human.

Western blotting

Rat left atria were homogenized and proteins were isolated as previously described (11). Soluble proteins were quantified using the Pierce™ BCA Protein Assay Kit (Thermo Scientific, Villebon-sur-Yvette, France) and aliquots of 10 µg were rapidly stored at -80°C until western blot analysis. Proteins were separated using 10% NuPAGE Bis-Tris gels (Life technologies®) and transferred to nitrocellulose membranes for saturation with 5% milk in TBS with 0.1% Tween 20 for one hour. After rinsing, membranes were incubated overnight at 4°C with primary antibodies in TBS with 0.1% Tween and 5% BSA. Membranes were then incubated at room temperature either for 90 minutes with horseradish peroxidase (HRP)-conjugated secondary anti-mouse and anti-rabbit antibodies (Life Technologies) or with biotinylated secondary anti-mouse and anti-rabbit antibodies followed by 30 minutes with streptavidin-HRP coupled antibodies to assess protein phosphorylation. Proteins were revealed using the enhanced chemiluminescence Plus method (Pierce) and Camera LAS 4000 (GE Healthcare). Relative protein levels were quantified by densitometry using the ImageQuant software (GE Healthcare).

Histochemistry

Just after sampling, atria were fixed into 10 % buffered formalin, dehydrated in 95 % ethanol and then in acetone, impregnated with methyl salicylate and embedded in paraffin. Collagen and muscle fibers were stained using Trichrome (Masson) kit according to the manufacturer's instructions (Sigma-Aldrich, St Quentin Fallavier, France). Picrosirius red staining was performed as previously described (12). Large images of whole atrial sections (eight µm) were acquired at magnification 100x and resolution of 1280 x 1024 pixels with a Nikon DS-Ri1 camera coupled to Eclipse-Ti Nikon microscope and Nis-Element software (Nikon France S.A., Champigny sur Marne, France). Subendothelial and interstitial collagen stained with picrosirius red (were quantified with Histolab software (Microvision Instruments, Evry, France), on three/four non-serial sections for each atria. Subendothelial and interstitial fibrosis are expressed as percentage of myocardial surface. For illustration of atrial remodeling and interstitial fibrosis, images were acquired at magnification 600x.

Epifluorescence microscopy

Left atria were fixed with paraformaldehyde (Sigma, St Quentin-Fallavier, France) for 15 minutes at room temperature, washed three times in PBS and immersed in 20% sucrose for 2h at 4°C. Left atria were then embedded in Cryomatrix™ resin (Thermo Electron SAS, Asnières, France), progressively frozen over liquid nitrogen, and stored at –80°C. Ten µm-thick cross-sections were permeabilized and saturated with 0.5% Triton X-100, 1% BSA and a 10% each of goat, chicken and rat serum in phosphate buffered saline (PBS) for 60 minutes. For staining of sarcolemmal membranes, atrial cross-sections were labeled overnight at 4°C with wheat germ agglutinin conjugated to Alexa Fluor® 488 (1/200) and DAPI (1/500) (Life Technologies) in PBS containing 1% BSA and 3% of rat serum Finally, glass slides with the labeled tissues were washed three-times in PBS and coverslips were mounted with Dako fluorescent mounting medium. Images were acquired at a magnification of ×600.
Atrial sections captures were performed on an Olympus Fluoview 1000 confocal microscope (Rungis, France). The myocytes diameters along the short-axis were measured for 15 cells/field and calculated using the Image J software. For each rat, the analysis was performed on two or three sections and five fields visualized each. Only myocytes with visible nuclei were analyzed.

Human tissue samples

Right and left atrial appendages were obtained from patients undergoing cardiac bypass surgery or valve repair at the Cardiology Institute of Hôpital Pitié-Salpêtrière (Paris, France) in accordance with

French legislation (L. 1211-3-9). Control patients never had any history of atrial fibrillation and no dilatation of right or left atria. The ten patients with paroxysmal AF had dilatation of both left and right atria. The baseline clinical characteristics of patients are shown in online table 2. To exclude the impact of co-variables on the results of PCR and western blot analysis, control and AF patients with left ventricular ejection fractions ≥ 45% were matched in regard to age, gender, comorbidity and medications excluding vitamin K antagonists (AVK) and anti-arrhythmic drugs.

Statistical analysis

Statistical analysis was performed using the Graph-Pad Prism 6 Software (GraphPad Inc.). The comparisons of three or more groups have been done with the non-parametric Kruskal-Wallis test, followed up with a series of pair-wise comparisons via the Mann-Whitney U-test. The dose-dependent effects of treatments have been analyzed with the non-parametric Kruskal-Wallis test, followed up with post-hoc multiple comparisons via the Dunn’s test. The comparison of the same group before and after treatment has been performed with the Wilcoxon matched-pairs signed-ranks test. The comparison of two different groups was analyzed via the Mann-Whitney U-test. The linear relationship between two variables was evaluated with the Pearson correlation.

References

1. Rupin A, Marx I, Vallez MO, et al. S35972, a direct-acting thrombin inhibitor with high oral bioavailability and antithrombotic efficacy. J. Thromb. Haemost. JTH 2011;9:1375–1382.
2. Perez M, Lamothe M, Maraval C, et al. Discovery of novel protease activated receptors 1 antagonists with potent antithrombotic activity in vivo. J. Med. Chem. 2009;52:5826–5836.
3. Vié B, Sablayrolles S, Létienne R, et al. 3-(R)-[3-(2-methoxyphenylthio-2-(S)-methylpropyl]amino-3,4-dihydro-2H-1,5-benzoxathiepine bromhydrate (F 15845) prevents ischemia-induced heart remodeling by reduction of the intracellular Na+ overload. J. Pharmacol. Exp. Ther. 2009;330:696–703.
4. Milliez P, Deangelis N, Rucker-Martin C, et al. Spironolactone reduces fibrosis of dilated atria during heart failure in rats with myocardial infarction. Eur. Heart J. 2005;26:2193–2199.
5. Sanchez C, Poggi M, Morange P-E, et al. Diet Modulates Endogenous Thrombin Generation, A Biological Estimate of Thrombosis Risk, Independently of the Metabolic Status. Arterioscler. Thromb. Vasc. Biol. 2012;32:2394–2404.
6. Cuker A, Siegal DM, Crowther MA, Garcia DA. Laboratory Measurement of the Anticoagulant Activity of the Non–Vitamin K Oral Anticoagulants. J. Am. Coll. Cardiol. 2014;64:1128–1139.
7. Korostelev M, Bihan K, Ferreol L, et al. Simultaneous determination of rivaroxaban and dabigatran levels in human plasma by high-performance liquid chromatography–tandem mass spectrometry. J. Pharm. Biomed. Anal. 2014;100:230–235.
8. Venteclef N, Guglielmi V, Balse E, et al. Human epicardial adipose tissue induces fibrosis of the atrial myocardium through the secretion of adipo-fibrokines. Eur. Heart J. 2015;36:795–805.
9. Chieng-Yane P, Bocquet A, Létienne R, et al. Protease-Activated Receptor-1 Antagonist F 16618 Reduces Arterial Restenosis by Down-Regulation of Tumor Necrosis Factor α and Matrix Metalloproteinase 7 Expression, Migration, and Proliferation of Vascular Smooth Muscle Cells. J. Pharmacol. Exp. Ther. 2011;336:643–651.
10. Brattelid T, Winer LH, Levy FO, Liestøl K, Sejersted OM, Andersson KB. Reference gene alternatives to Gapdh in rodent and human heart failure gene expression studies. BMC Mol. Biol. 2010;11:22.
11. Boycott HE, Barbier CSM, Eichel CA, et al. Shear stress triggers insertion of voltage-gated potassium channels from intracellular compartments in atrial myocytes. Proc. Natl. Acad. Sci. U. S. A. 2013;110:E3955–3964.
12. Mougenot N, Bos R, Médiani O, Lechat P. Captopril–furosemide survival study in experimental heart failure. Fundam. Clin. Pharmacol. 2005;19:457–464.

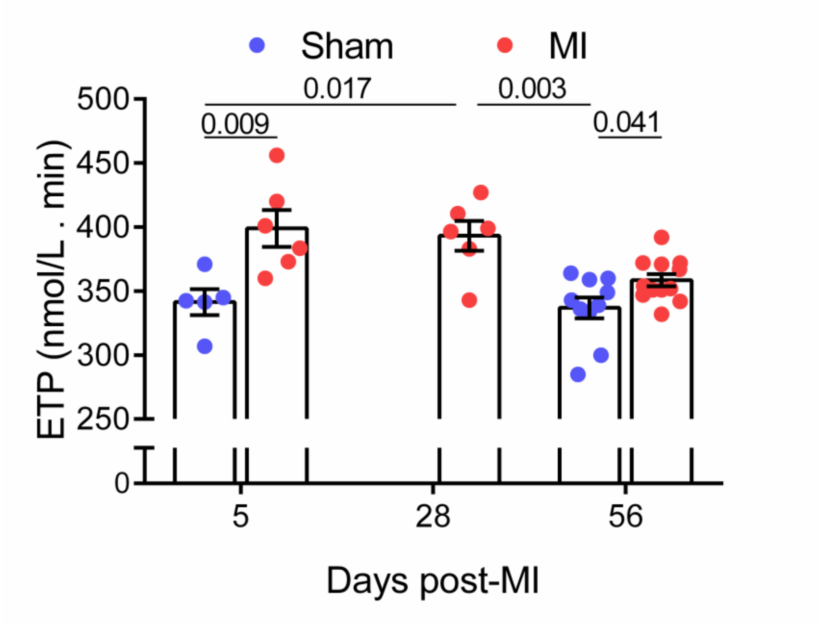

Online Figure 1: MI-induced heart failure triggers a thrombogenic potential in plasma Endogenous thrombin potential (ETP) was measured in platelet-poor plasma from blood collected at different times following MI. N=5-12 for each group of rats. The exact p-values indicated on the figure are calculated *vs* sham at corresponding time.

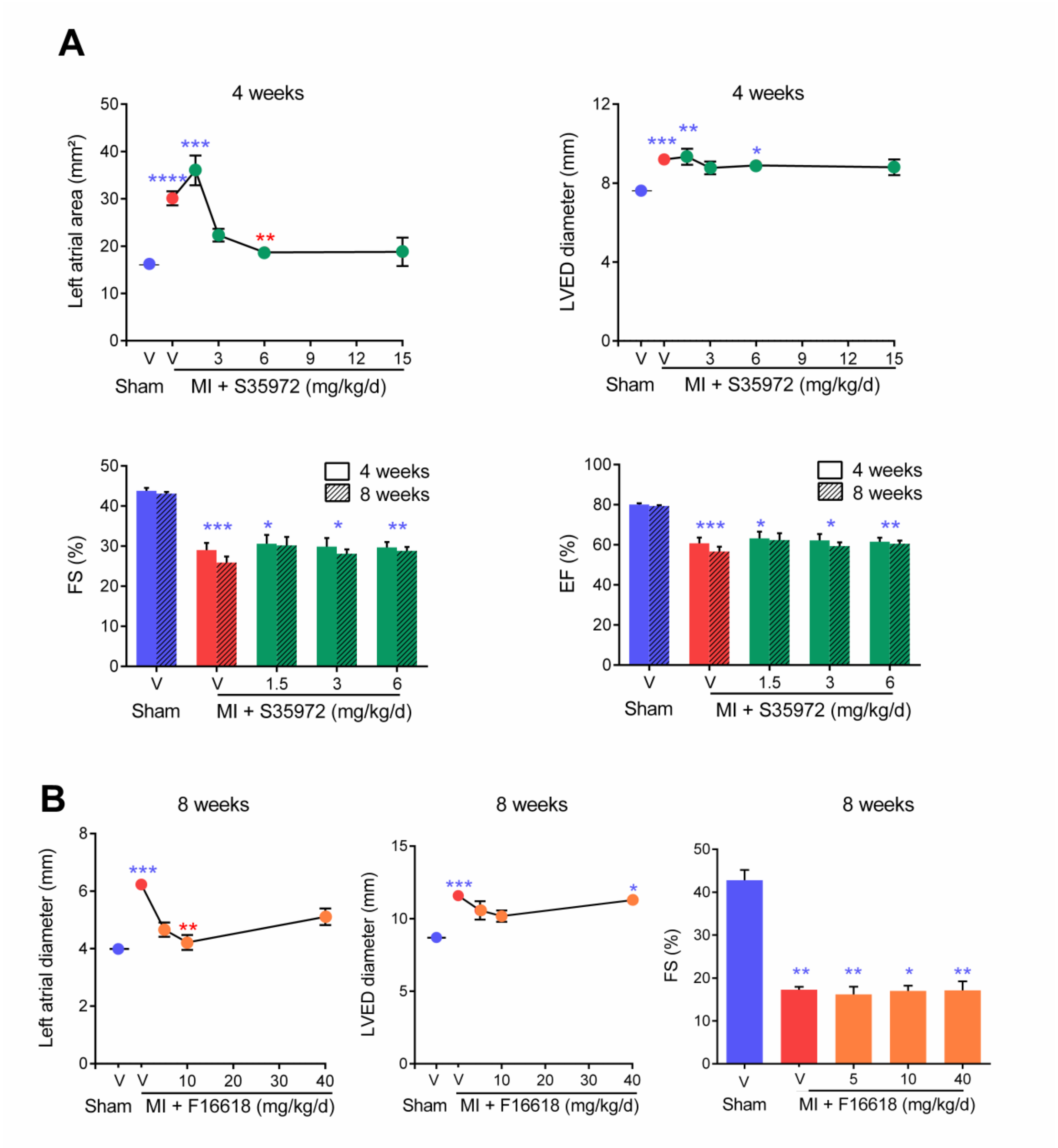

Online Figure 2: Treatment with DTI or PAR-1 antagonist did not improve the left ventricle function. (**A**) DTI S35972 or vehicle (V: saline) was subcutaneously delivered with mini-pumps for four and eight weeks. (**B**) PAR-1 antagonist F16618 or vehicle (V: 1% methylcellulose) was administered once-daily by gavage for eight weeks. Left atrial area was assessed by echocardiography in B-mode and left ventricle end-diastolic (LVED) diameters, fractional shortening (FS), ejection fraction (EF) in TM-mode. Data are Means ± SEM. N= 10-12 and 14-15 for vehicle-treated sham and MI-rats and 5-10 for each dose, **P*<0.05, ***P*<0.01 *** *P*<0.001 and **** *P*<0.0001 *vs* sham in red and *vs* vehicle-treated MI-rats in red.

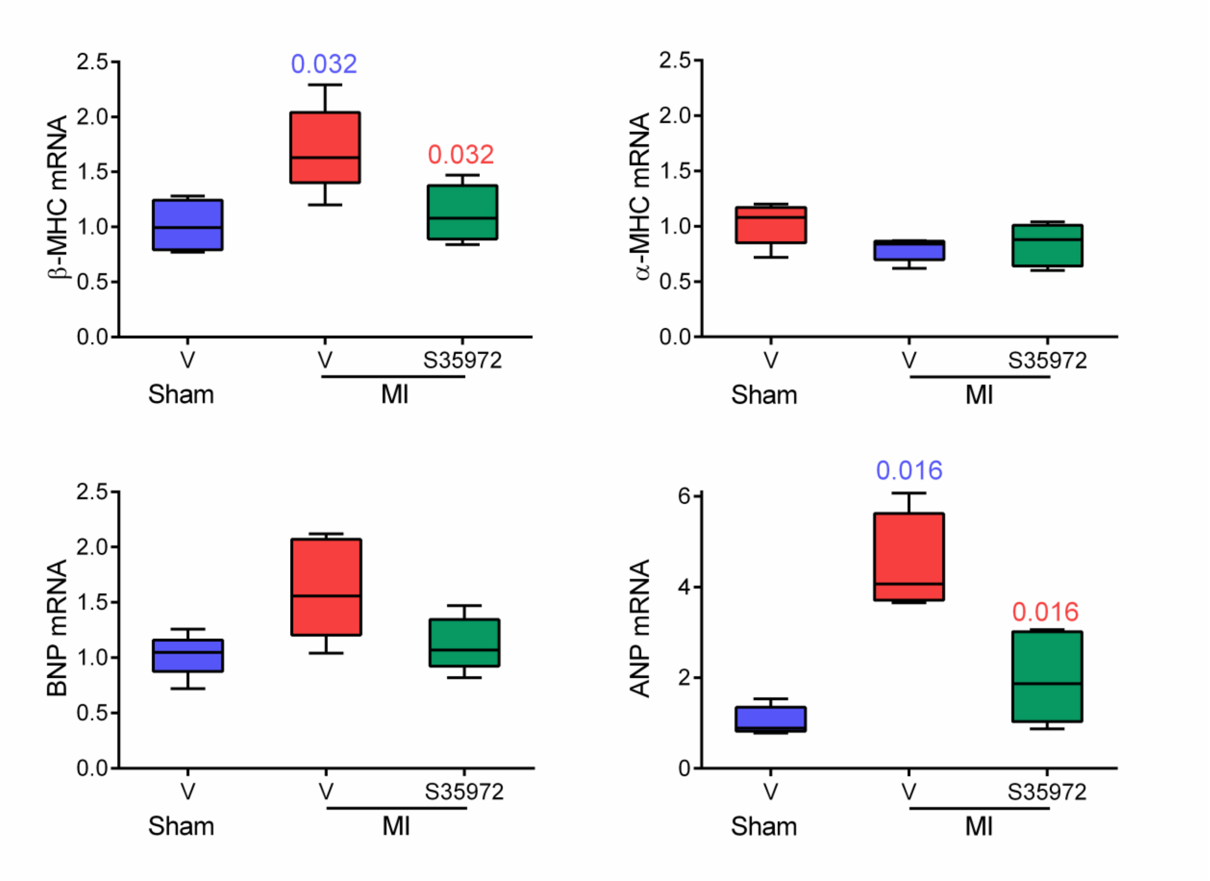

Online Figure 3: Treatment with DTI S35972 reduced the overexpression of hypertrophic biomarkers in left ventricle from MI-rats. After the surgical MI, ALZET® osmotic minipumps were subcutaneously implanted for continuous delivery of 6 mg/kg/d S35972 or vehicle (V: saline) for eight weeks. Relative expression of mRNAs for β and α isoforms of myosin heavy chain (MHC), brain and atrial natriuretic peptide (BNP and ANP) was analyzed by real-time PCR. N=5 for each group. The exact p-values calculated *vs* sham (blue) or*vs* vehicle-treated MI-rats (red) are indicated when significant.

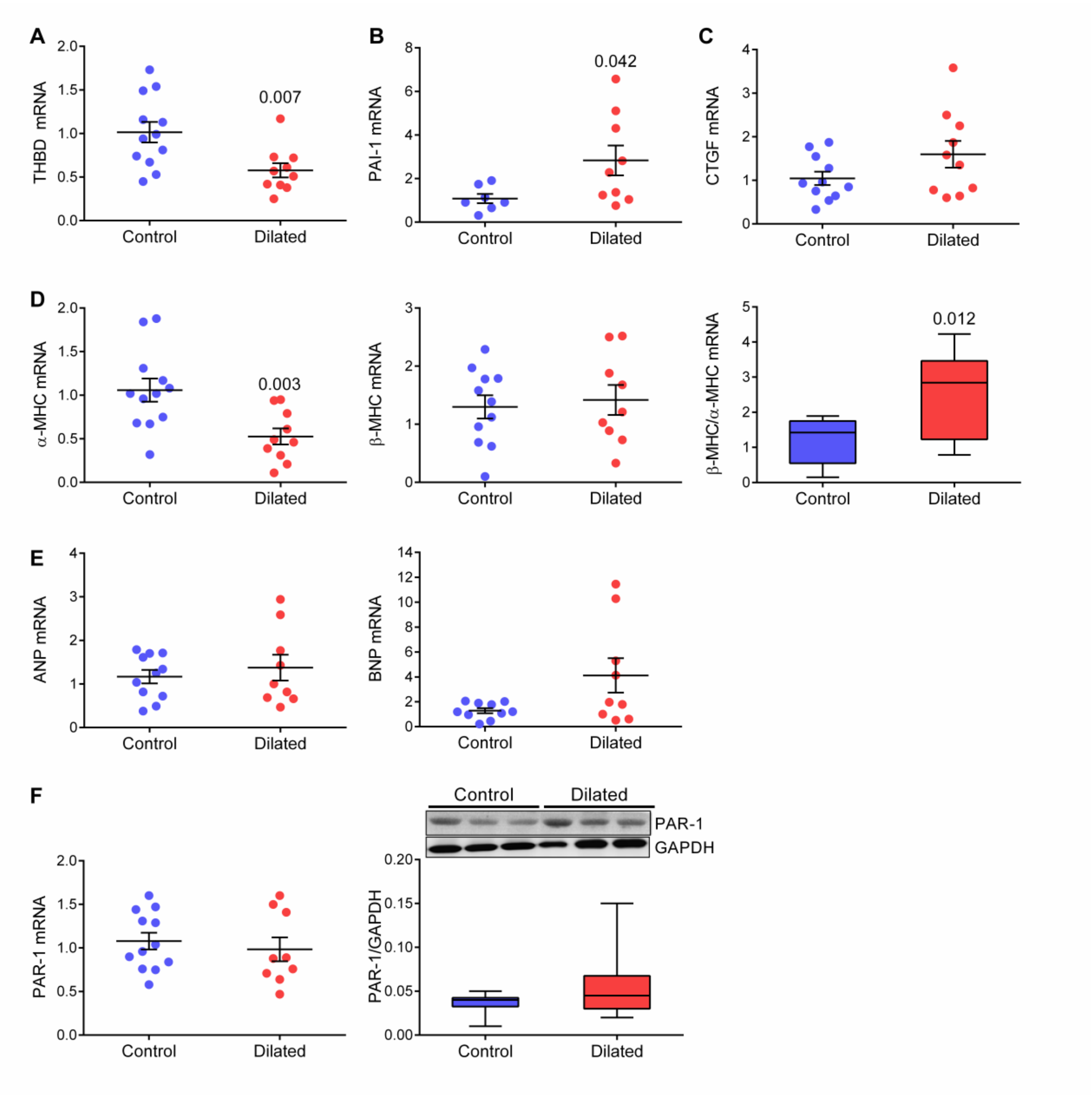

Online Figure 4: Phenotypic modifications in dilated atria from patients with paroxysmal atrial fibrillation. Left and right atria were sampled during cardiac surgery from patients with non-dilated atria and patients with right and left dilated atria and paroxysmal atrial fibrillation (Dilated). Analysis by real-time PCR of relative mRNA expression for thrombomodulin (THBD, **A**) plasminogen activator inhibitor 1 (PAI-1, **B),** connective tissue growth factor (CTGF, **C**), α and β isoforms of myosin heavy chain (MHC) and β/α ratio (**D**), atrial and brain natriuretic peptides (ANP and BNP, **E**), and protease activated receptor 1 (PAR-1, **F**). (**F**, right panel) Representative immunoblots and histograms of densitometric analysis of PAR-1 protein. N=7-10 for dilated group and 7-12 for control. The exact p-values calculated *vs* Control are indicated when significant.

Online Table 1: Gene-specific primers for rat atria samples (5'➔3')

Protein	Gene	sense	antisense
PAI-1	*SERPINE-1*	AGTCTTTCCGACCAAGAGCA	GCGGGCTGAGACTAGAATGG
CTGF	*CCN2*	CAGGGAGTAAGGGACACGAA	CAGTCTTCCAGGAGGCTCAC
PAR-1	*F2R*	AGAAGTCGCGGGCTTTGTT	AGGACTGTCGGAGAGGAGCAG
α-MHC	*MYH6*	TCAGAG-AAGGAGCGCCTAGA	GGTCCTCCTTCACAGTCACC
β-MHC	*MYH7*	ACAGAGGAAGACAGGAAGAACCTACTG	GCACAAGATCTACTCCTCATTCAGG
ANP	*NPPA*	GATTTCAAGAACCTGCTAGACCA	CTCTCTGAGACGGGTTGACTT
BNP	*NPPB*	CAATCCACGATGCAGAAGCTG	CGCTGTCTTGAGACCTAAGGACT
RPL32	*RPL32*	CCAGAGGCATCGACAACA	GCACTTCCAGCTCCTTGA
Rplp0	*RPLP0*	CGACCTGGAAGTCCAACTAC	GTCTGCTCCCACAATGAAG
PolR2a	*POLR2A*	CGTATCCGCATCATGAACAGTGA	TCATCCATCTTATCCACCACCTCTT

Online Table 2: Baseline patient characteristics

Characteristics	Control (n=12)	Paroxysmal AF (n=10)	*P**
Age, y	60.5±11.4	60.0±16.2	0.884
Gender, male/female	8/4	5/6	0.391
Diabetes	6	2	0.204
Dyslipidemia	8	3	0.198
Hypertension	8	4	0.396
Smoking	6	4	0.691
Myocardial Infarction	4	2	0.338
Left ventricle ejection fraction (%)	60.0±6.8	61.5±11.7	0.755
Medical therapies			
Beta-blockers	10	4	0.074
ACE inhibitor/ARB	7	3	0.230
Anti-platelets agents	7	5	0.754
VKA	0	7	0.0007
Anti-arrhythmic drugs	0	6	0.0096
Type of heart surgery			
CABG	8	0	
Mitral valve repair	0	10	
Aortic valve repair	5	2	
Tricuspid valve repair	0	4	

ACE, Angiotensin converting enzyme; ARB, Angiotensin receptor blocker; VKA, vitamin K antagonist; CABG, coronary artery bypass grafting. Values are mean ± SD or n. Percentages were calculated according to the available data. * Mann-Whitney rank sum test was used to compare the two groups.

Online Table 3: Gene-specific primers for human atria samples (5'➔3')

Protein	Gene	sense	antisense
PAI-1	*serpine1*	AGCTCCTTGTACAGATGCCG	ACAACAGGAGGAGAAACCCA
CTGF	*ctgf*	TACCAATGACAACGCCTCCT	TGGAGATTTTGGGAGTAC
PAR-1	*f2r*	CTGTGTACACCGGAGTGTTTGTA	GATCTTAAAGGGGAGCACAGA
α-MHC	*myh6*	ATTGCTGAAACCGAGAATGG	CGCTCCTTGAGGTTGAAAAG
β-MHC	*myh7*	ACCTGGAGAATGACAAGCAG	CCTGTTCATCCTCAATCCTT
ANP	*nppa*	CTGATGGATTTCAAGAATTTGCTGG	TCATTCGGCTCACTGAGCACTT
BNP	*nppb*	TACAGGAGCAGCGCAACCATT	CCGCCTCAGCACTTTGCAG
Thrombomodulin	*thbd*	GCTACCCTAACTACGACCTGGT	CGCAGAGGTAGCTAGTTTGGTT
RPL32	*rpl32*	TAGAAGATTCAAGGGCCAGA	GGTTCTTGGAGGAAACATTG
RPL4	*rpl4*	CAGCACTGGTCATGTCTAAAGGT	AGCCTTCAACTTTATCTTCAACTACCAAA
PPIA	*ppia*	CCGAGGAAAACCGTGTACTA	CTGTCTTTGGGACCTTGTCT
